# NMR and MALDI-TOF MS based characterization of exopolysaccharides in anaerobic microbial aggregates from full-scale reactors

**DOI:** 10.1038/srep14316

**Published:** 2015-09-22

**Authors:** Graciela Gonzalez-Gil, Ludivine Thomas, Abdul-Hamid Emwas, Piet N. L. Lens, Pascal E. Saikaly

**Affiliations:** 1 King Abdullah University of Science and Technology, Biological and Environmental Sciences and Engineering Division, Water Desalination and Reuse Center. Thuwal 23955–6900, Saudi Arabia; 2 UNESCO-IHE, Westvest 7, 2611 AX Delft, the Netherlands; 3 King Abdullah University of Science and Technology, Biosciences Core Laboratory, Thuwal, Saudi Arabia; 4 King Abdullah University of Science and Technology, Advanced Nanofabrication Imaging and Characterization, Thuwal, Saudi Arabia

## Abstract

Anaerobic granular sludge is composed of multispecies microbial aggregates embedded in a matrix of extracellular polymeric substances (EPS). Here we characterized the chemical fingerprint of the polysaccharide fraction of EPS in anaerobic granules obtained from full-scale reactors treating different types of wastewater. Nuclear magnetic resonance (NMR) signals of the polysaccharide region from the granules were very complex, likely as a result of the diverse microbial population in the granules. Using nonmetric multidimensional scaling (NMDS), the ^1^H NMR signals of reference polysaccharides (gellan, xanthan, alginate) and those of the anaerobic granules revealed that there were similarities between the polysaccharides extracted from granules and the reference polysaccharide alginate. Further analysis of the exopolysaccharides from anaerobic granules, and reference polysaccharides using matrix-assisted laser desorption/ionization time-of-flight mass spectrometry (MALDI-TOF MS) revealed that exopolysaccharides from two of the anaerobic granular sludges studied exhibited spectra similar to that of alginate. The presence of sequences related to the synthesis of alginate was confirmed in the metagenomes of the granules. Collectively these results suggest that alginate-like exopolysaccharides are constituents of the EPS matrix in anaerobic granular sludge treating different industrial wastewater. This finding expands the engineered environments where alginate has been found as EPS constituent of microbial aggregates.

The capacity of microorganisms to form aggregates (i.e., granules) has been the key to the development of high-rate wastewater treatment reactors such as the up-flow and expanded anaerobic granular sludge bed (UASB and EGSB) reactors. Currently, various types of microbial granular communities are being used to treat diverse types of industrial and domestic wastewaters under anaerobic, aerobic or anoxic conditions^1^,^2^.

Granular sludge is composed of multispecies microbial consortia embedded in a matrix of inorganic and extracellular polymeric substances (EPS). Polysaccharides and proteins are the main constituents of EPS, with minor quantities of extracellular DNA, humic acids and lipids^3^,^4^. Characterizing the EPS fraction of granules may lead to understanding the relationships between microorganisms present therein and the EPS composition as well as their role in granules mechanical stability^5^, a paramount feature for the stable operation of granular sludge-based reactors. The content and the constituents of the EPS from granular sludge may vary^6^,^7^. This variation has been attributed to factors such as microbial growth phase, nitrogen and phosphorus availability, type of carbon source, sludge retention time and shear stress, to name a few^7^,^8^.

Exopolysaccharides have been considered relevant in microbial aggregation since some can form viscous or gel-like matrices in which microorganisms can be embedded or attached^9^. Despite their relevance in microbial aggregation, few exopolysaccharides from granular sludge systems have been characterized, and studies regarding the relationship between the microbial community structure and the nature of exopolysaccharides are scarce. The chemical structure of an exopolysaccharide from a laboratory-scale aerobic granular sludge reactor fed with acetate was elucidated as a complex heteropolysaccharide using nuclear magnetic resonance (NMR)^10^, and its production was linked to the dominance of a single bacterial type in the sludge^11^. However, the main exopolysaccharide of aerobic granules cultivated in a pilot plant treating a mixture of municipal sewage and abattoir wastewater seemed to have a similar chemical structure to seaweed alginate, as revealed by matrix-assisted laser desorption/ionization time-of-flight mass spectrometry (MALDI-TOF MS)^12^. This alginate-like exopolysaccharide was proposed to be responsible for maintaining the gel-like structure of those aerobic granules; the granules microbial community structure was not investigated though.

In contrast to aerobic granules, the nature of the exopolysaccharides from anaerobic granules has not been investigated before using advanced techniques such as NMR and MALDI-TOF. Most studies in anaerobic granules have focused on bulk EPS characterizations describing EPS absorbance and or fluorescence properties^13^,^14^ and studies that simultaneously characterize the exopolysaccharides and the microbial communities are non-existent. Since the phenomenon of granulation is independent of aerobic or anaerobic environments and since granulation occurs in reactors treating different types of wastewaters, the following questions were raised: i) is there a common exopolysaccharide in anaerobic granules treating industrial wastewaters, ii) whether alginate-like exopolysaccharides are also present in anaerobic granules, and iii) whether the presence of certain exopolysaccharide is linked to a particular microbial community. Therefore, the aim of this study was to get insight into the chemical fingerprint of the exopolysaccharide fraction of EPS in anaerobic granules obtained from various full-scale reactors using NMR and MALDI-TOF MS approaches. 16S rRNA amplicon and metagenomic pyrosequencing were further used to characterize the microbial composition of the anaerobic granules and to investigate the presence of genes related to exopolysaccharides metabolism.

## Results and Discussion

### ^1^H NMR Fingerprint of Extracellular Polysaccharides of Anaerobic Granules

**Figure 1:**
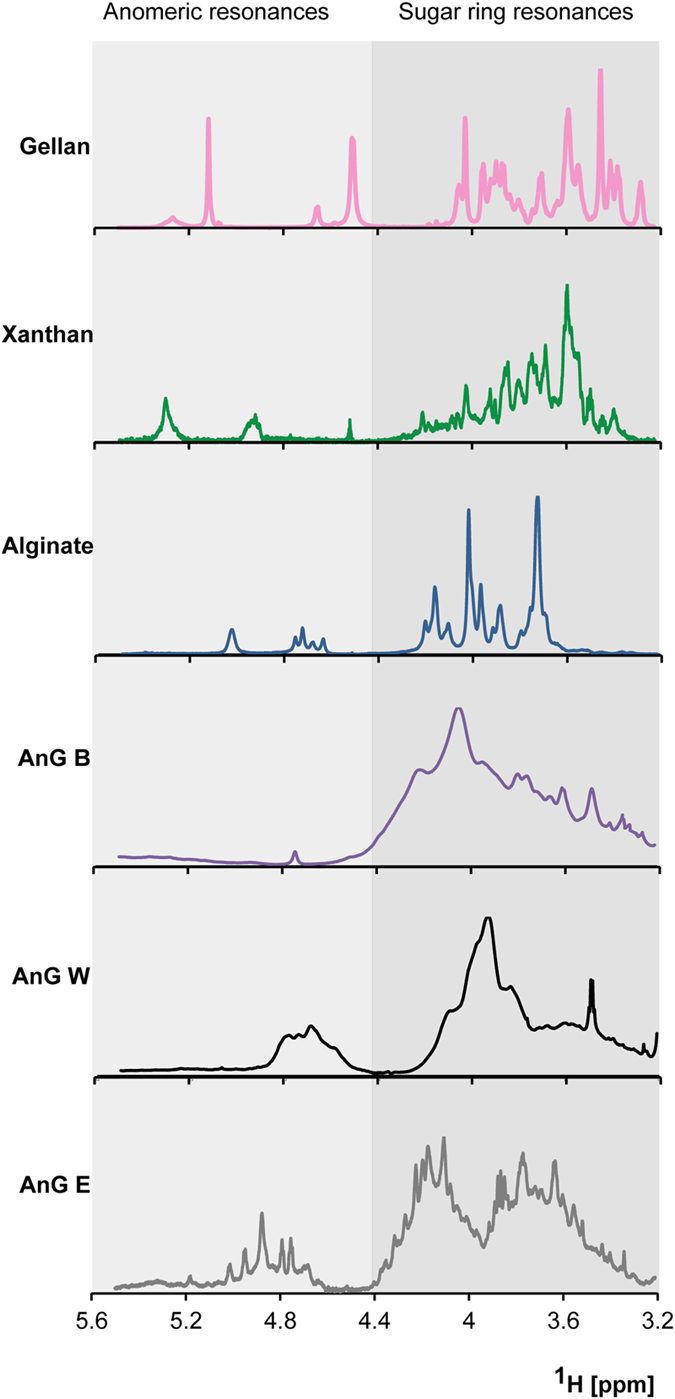
^1^H spectra of reference exopolysaccharides (gellan, xanthan, alginate) and exopolysaccharides extracted from various anaerobic granules (AnG).

The ^1^H NMR spectrum of polysaccharides can be divided into regions containing resonances corresponding to the sugar ring protons (3.2 to 4.4 ppm) and the anomeric protons (4.4 to 5.6 ppm)^15^. The ^1^H NMR spectra of the extracted exopolysaccharides from anaerobic granules displayed sugar ring resonances, at 3.2 to 4.4 ppm, that are typical of exopolysaccharides such as gellan, xanthan and alginate (Fig. 1). In the region corresponding to the anomeric resonances (4.5 to 5.1 ppm), the four peaks observed at 4.6 to 4.8 ppm in the alginate spectrum (Fig. 1) have been previously assigned to the proton resonances of guluronic (G) and manuronic (M) acids, which are the two building blocks of alginate^16^. These monomers may be arranged in GG, MM or GM blocks distributed in a non-regular, block-wise order along the alginate chain^16^. In this same anomeric region, some peaks were also observed in the granule spectra, but these were not as clearly resolved as those of the alginate. It was reported that particularly G-block associations, through calcium binding, can result in less resolved ^1^H NMR resonances^17^. Considering that alginate may be part of the exopolysaccharide signal of the granule samples, determining the proportion of the G and M building blocks would require well resolved peaks which may be achieved using a controlled depolymerization pretreatment^16^. Thus, this aspect should be addressed in further investigations. In addition to the polysaccharides resonances, the samples also showed resonances below 3.2 ppm (Figure S1), which are characteristics of proteins and lipids^10^, and acetyl and succinyl groups as observed in exopolysaccharides from marine bacteria^18^. The efficiency of various acids to precipitate proteins differs^19^. It appears that not all proteins were removed from the polysaccharide fraction of the anaerobic granules using perchloric acid. Similarly, resonances below 3.2 ppm were detected in the polysaccharide fraction of aerobic granules when using the same acid to remove proteins^10^. However, in that study, when the polysaccharide fraction was further enriched by fractional precipitation with cetylpyridinium chloride and methanol, the number of resonance signals below 3.2 ppm was reduced, and gel permeation chromatography was additionally used to completely remove proteins^20^. Thus, further studies on anaerobic granules should investigate the use of this polysaccharide enrichment approach.

Despite that NMR can be a powerful tool to elucidate the chemical structure of compounds, the extensive resonance overlap when probing complex mixtures of compounds can hamper structural analysis. Also, the ^1^H NMR signals of the exopolysaccharides extracted from the granular sludges in this study proved to be complex, likely as a result of the diverse microbial population in the granules as shown in the microbial community section below. In this study, the amount of sample was not sufficient to conduct ^13^C NMR experiments, which are used for detailed elucidation of chemical structures. On the contrary, the chemical structure of a exopolysaccharide produced by laboratory-scale aerobic granules was elucidated by Seviour *et al.*^10^ using a set of ^1^H and ^13^C NMR experiments. In the aerobic granules of that study, a single high molecular weight heteropolysaccharide, composed of eight different sugar residues and named “granulan” by the authors, was the dominant exopolysaccharide in the EPS extracts.

**Figure 2:**
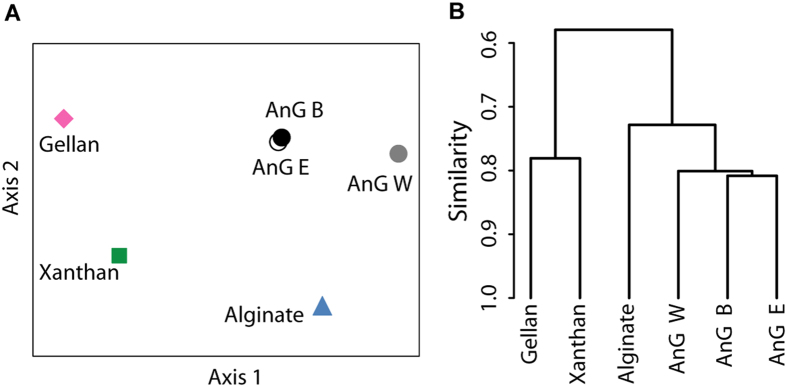
(**A**) Similarities between the ^1^H NMR spectra of reference polysaccharides and polysaccharides extracted from various anaerobic granules (AnG) represented by non-parametric multidimensional scaling. Stress = 0.07; values < 0.1 indicate an acceptable representation of the structure of the data set by the ordination^42^. (**B**) Hierarchical clustering of the ^1^H NMR spectra of reference and granular sludge polysaccharides based on Bray-Curtis similarity. The cophenetic correlation coefficient, which indicates the validity of the cluster information, was 0.88; values higher than 0.8 are considered to represent well the pattern of similarities^43^,^44^.

In addition to elucidating the chemical structure of compounds, the ^1^H NMR spectra could also be used as chemical fingerprints, where spectral regions of interests for various samples may be compared. Therefore, similarities in ^1^H NMR spectra comprising the ring and anomeric region of the polysaccharides (3.2 to 5.6 ppm) obtained for the different granules were visually represented using NMDS analysis. Represented in two-dimensions, the exopolysaccharide fingerprints of all the granules were relatively close together (Fig. 2). Furthermore, the exopolysaccharides fingerprints of all the granules cluster closer to the fingerprint of the reference polysaccharide alginate than to the fingerprints of the other reference polysaccharides (Fig. 2). Although the number of samples analyzed in this study was limited, the use of ^1^H NMR, combined with NMDS could potentially be used as a method for monitoring or comparing profiles of exopolysaccharides from complex samples such as those of granular sludges. For example, it can be tested if the type of wastewater defines the production of certain exopolysaccharides by capturing the ^1^H NMR fingerprint of granular sludge from diverse industrial wastewater types.

One drawback of NMR is that due to the low natural abundance of ^13^C (≈1%), a large amount of sample is required to obtain ^13^C NMR signals necessary to characterize the chemical structure of complex molecules. The low EPS content of the granular sludge of this study (Table S1) hampered further NMR characterization (i.e., ^13^C NMR-based characterization). Therefore, MALDI-TOF MS was tested as an alternative approach to further characterize the exopolysaccharides of the granular sludges.

### MALDI-TOF MS Fingerprint of Extracellular Polysaccharides of Anaerobic Granules

**Figure 3:**
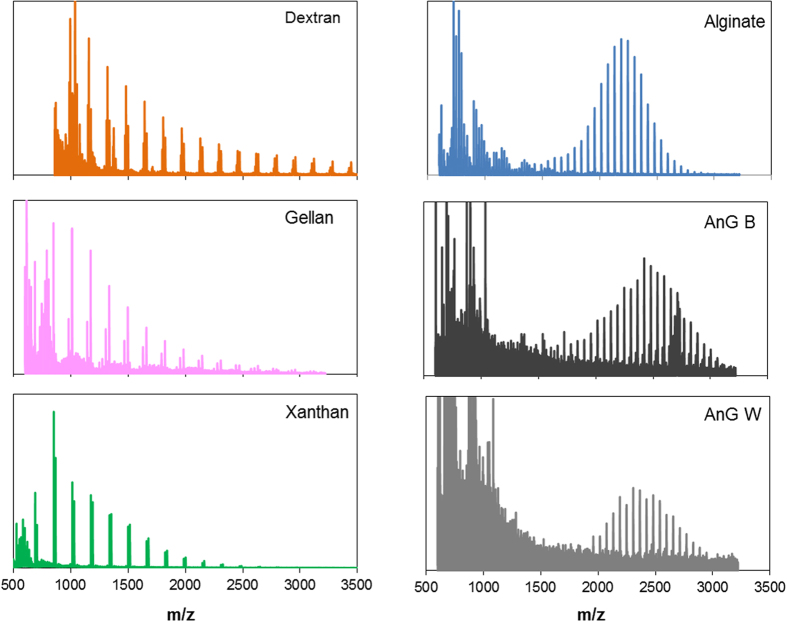
MALDI-TOF MS spectra of reference polysaccharides and of exopolysaccharides from anaerobic granules AnG B and AnG W.

MALDI-TOF MS can be considered as a rapid and sensitive approach for obtaining fingerprints of polysaccharides^21^. All the reference polysaccharides exhibited spectra that could be distinguished from each other (Fig. 3). Dextran, gellan and xanthan are polysaccharides produced mainly by *Lactobacillus*, *Sphingomonas* and *Xanthomonas*, respectively^22^. The latter two bacterial genera have been found in wastewater treatment systems^23^. The use of a reference (chemically well characterized) exopolysaccharide from anaerobic microorganisms inhabiting anaerobic granules was not possible since currently there is none available. Since an alginate-like exopolysaccharide has been found in aerobic granules^12^, alginate was also used as reference exopolysaccharide in this study.

**Figure 4:**
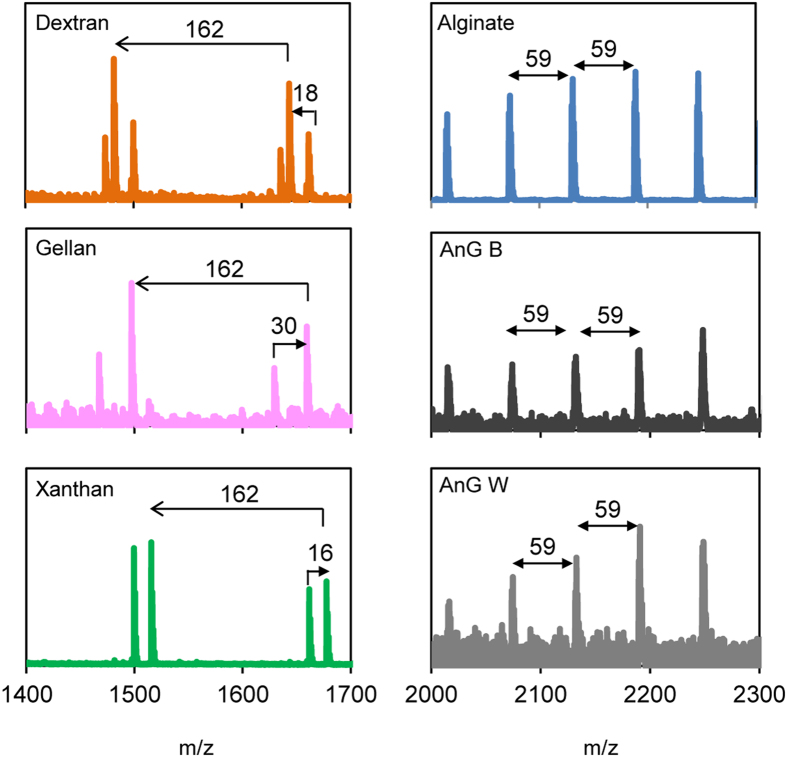
Zoomed view of spectra from panel (A). For dextran, gellan and xanthan the satellite peaks of the hexose residues (162 m/z) are shown. For alginate and the exopolysaccharides of anaerobic granules AnG B and AnG W, part of the bell-shape peak series having 59 m/z intervals is shown.

Dextran, gellan and xanthan, showed serial peaks with mass unit intervals of 162 m/z, which is consistent with hexose unit residues (Figs 3 and 4). However, spectra of these reference polysaccharides can still be distinguished from each other by examining their satellite signals (Fig. 4). Satellite peaks are peaks close to or shouldering main peaks in spectra. Dextran showed satellite peaks of 18 m/z above each molecular ion (i.e., hexose unit) (Fig. 4), consistent with the loss of water (Figure S2). The satellite peaks of gellan were 30 m/z below each molecular ion (Fig. 4), which is probably due to the loss of an aldehyde group from the glucose unit (Figure S2), whereas the satellite peaks of xanthan were 16 m/z above each molecular ion (Fig. 4) which may be consistent with the loss of an oxygen atom (Figure S2).

**Figure 5:**
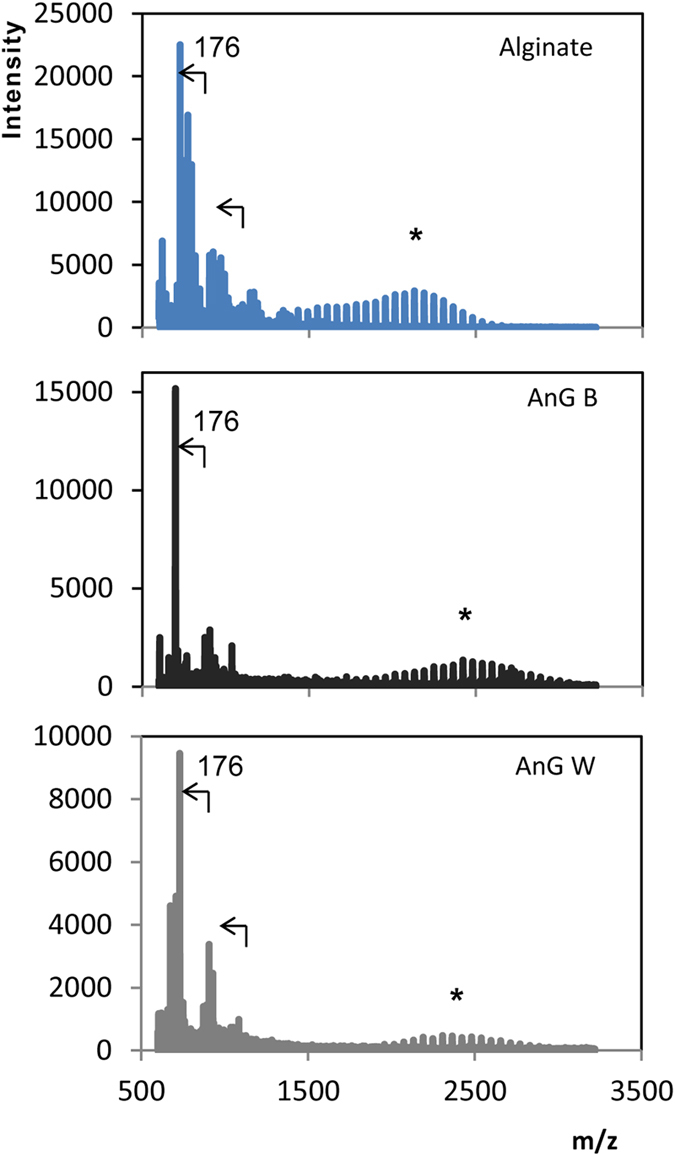
MALDI-TOF MS spectra of alginate and of exopolysaccharides from anaerobic granules AnG B and AnG W exhibiting alginate-like signals. Arrows indicate m/z differences of 176. The asterisks indicate the bell-shaped zone of the spectra shown in detailed in Fig. 3.

In contrast, the spectra of alginate reproducibly exhibited a distinct bell-shape series of peaks (Fig. 3) with a peak-to-peak difference of 59 m/z (Fig. 4). Regular repetitive signal patterns are typical of polymers. Similar results were obtained using the α-cyano-4-hydroxycinnamic acid (HCCA) matrix, another matrix often used in oligosaccharide analyses (data not shown). Two of the granular sludges investigated, AnG B and AnG W, reproducibly showed a MALDI-TOF MS spectrum that resembled the distinct bell-shape spectrum of the reference alginate (Fig. 3). The peak-to-peak difference of these granular sludge exopolysaccharides was also 59 m/z (Fig. 4), which corresponds to the molecular weight of an acetyl group, thus it would suggest de-acetylation of the acetylated mannuronate residues of alginate (Figure S2). According to the manufacturer, the reference alginate originates from a brown alga. Considering that a main difference between algal and bacterial alginate is that the latter is O-acetylated while algal alginate is not,^9^ it is thus likely that a fragmentation mechanism^24^ other than de-O-acetylation occurs during the analysis of the reference alginate and alginate-like exopolysaccharides from the anaerobic sludge samples. The exact mechanism deserves further investigation though. The bell-shaped signal was only detected with the alginate and with the alginate-like exopolysaccharides of granules AnG B and AnG W. In the same spectra, peak-to-peak signals of 176 m/z corresponding to alginate units (i.e. guluronic acid residues) were also detected (Fig. 5), thus further suggesting the presence of alginate in these anaerobic granular sludges. It should be noted that MALDI-TOF does not merely reflect the presence of a certain unit or moiety in an analyte. For example, similar to alginate, xanthan and gellan also contain uronic acid units in their structure (the chemical structure of these polysaccharides is shown in Figure S2). However the chemical structure of xanthan and gellan differs from that of alginate (Figure S2) and xanthan and gellan ionization occurred in the glucose residue (i.e., peak to peak signals 162 m/z) and not in the uronic acid (i.e., peak to peak difference of 176 m/z as observed in alginate), thus resulting in distinct MALDI-TOF MS spectra for these polysaccharides (Figs 3 and 5). This illustrates that although different polysaccharides share uronic acids in their structure, MALDI-TOF signals can differ as a result of differences in their chemical structure. Other polysaccharides containing uronic acids are of animal (mostly human) or plant origins^25^, and MALDI-TOF MS signals of the latter differ from alginate signals^26^. Therefore it is unlikely that these polysaccharides are produced in anaerobic granules. Note that the distinct bell-shaped signal of the alginate and the alginate-like exopolysaccharides from the granular sludges was not detected when using matrix alone or when directing the laser on the bare MALDI-TOF target plate (data not shown). NMDS may also be used to visually explore similarities of MALDI-TOF MS spectra of the exopolysaccharides (see Supporting information). As expected from the results in Figs 3 and 5, the spectra of the exopolysaccharides from the AnG B and AnG W samples were most similar to the spectrum of alginate (Figure S3).

The polysaccharides from the AnG E granules (paper mill wastewater) yield some signals (Figure S4). However, contrary to the spectra of the other granular samples, the spectra of the AnG E granules were not consistently reproduced. It is possible that the polysaccharides from these granules are of very high molecular weight or their oligomers stick together forming very large molecular aggregates that cannot be readily ionized by MALDI-TOF^27^. In this case, provided sufficient sample is available, hydrolysis and derivatization would be recommended.

**Figure 6:**
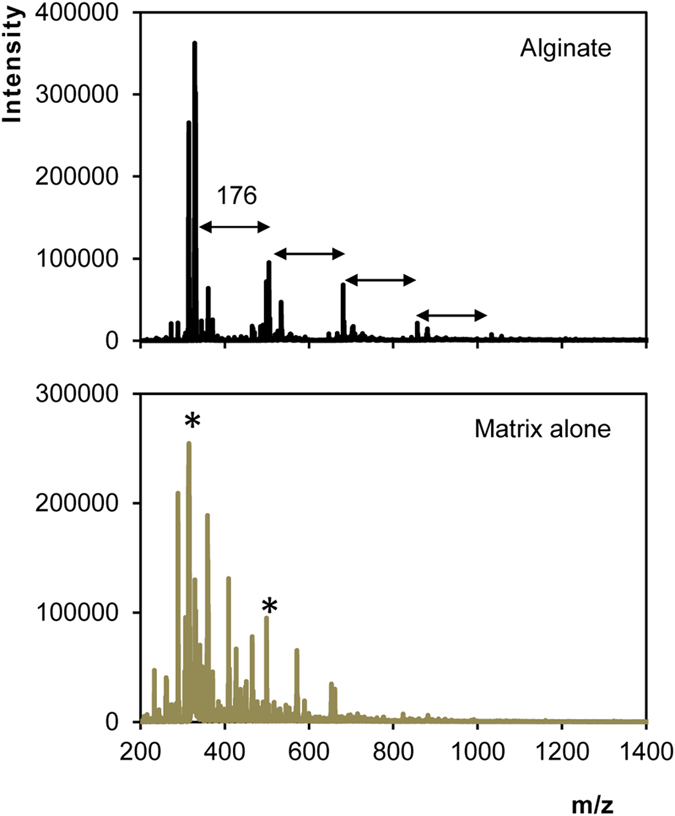
Negative mode of the MALDI-TOF MS spectrum of reference alginate and of the DHB matrix alone. The spectrum of the matrix alone shows peaks, indicated by the asterisk, which matched those of the alginate spectra.

An alginate-like polysaccharide extracted from aerobic granules was characterized by Lin *et al.*^12^ using MALDI-TOF MS. Their procedure involved de-acetylation of the polysaccharide prior to MALDI-TOF analysis and spectrum acquisition in negative mode. Initially in the present study, and following the procedure by Lin *et al.*^12^, the reference alginate was characterized in negative mode of MALDI-TOF MS. In accordance to the results of Lin *et al.*^12^, the negative mode of the alginate spectrum showed serial peaks with mass unit intervals of 176 m/z (Fig. 6), which is consistent with guloronic acid residues. However, for the remainder of the experiment, the positive ion mode was used because: (i) the spectrum of the DHB matrix alone in the negative mode displayed some peaks that matched some of those in the spectrum of the alginate in the DHB sample (also giving intervals of 176 m/z) (Fig. 6), (ii) the negative mode spectra were less consistent/reproducible compared to the positive, and (iii) the other reference exopolysaccharides did not yield meaningful spectra in the negative mode (data not shown).

In the current study, the polysaccharides were not pre-treated (e.g., partially hydrolyzed or derivatized) prior to their detection. Pre-treatment steps can be time consuming and laborious^21^,^28^. Although two of the granular sludges studied exhibited a distinct alginate signal, it is possible that other polysaccharides were also present but could not be ionized. Thus, additional investigations comparing pre-treated and non-pretreated samples are advisable.

### Microbial community and genes related to exopolysaccharide metabolism in the anaerobic granular sludges

In this study, 16S rRNA gene pyrosequencing was used to characterize the bacterial and archaeal community in the anaerobic granules (see Supporting information and Fig S5).

Although the most abundant bacterial class differed among the three granular sludge samples, the exopolysaccharides NMR fingerprints of all the granules clustered together, and were closer to the fingerprint of the reference polysaccharide alginate than to the fingerprints of the other reference polysaccharides (Fig. 2), suggesting that the exopolysaccharides in these granules are not expressed by a particular microbial population. Seivour *et al.*^11^ suggested that the exopolysaccharide “granulan”, was produced by a single bacterial type (*Candidatus* Competibacter) whose abundance was ≥60% in aerobic granules from an acetate fed laboratory-scale reactor. However, Lin *et al.*^12^ reported that an alginate-like exopolysaccharide was the main exopolysaccharide of aerobic granules cultivated in a pilot plant treating a mixture of municipal and abattoir wastewater. The difference between the two studies could be due to the different type of wastewater used (i.e. acetate *vs* municipal-abattoir water). However, a larger data set comprising microbial and exopolysaccharides fingerprints of full-scale granular sludge from diverse wastewater types would be necessary for recognition of patterns and data mining at higher resolution.

An unexplored issue is to what extent methanogens, abundant in anaerobic granules^29^, contribute to exopolysaccharide production. The abundance of archaea in the anaerobic granules, based on quantitative-PCR, was about 10 to 14% of the microbial community. Most of the methanogens were affiliated to the classes *Methanobacteria* and *Methanomicrobia* which comprise families of hydrogenotrophic and acetoclastic methanogens, respectively (Figure S5). Considering that the free energy change in methanogenesis is very low^30^, it is unlikely that methanogens contribute significantly to the production of exopolysaccharides. Morgan *et al.*^31^ proposed that methanogens are likely restricted in exopolysaccharide production because they lack a carrier molecule involved in the release of exopolysaccharides. However, studies on exopolysaccharide production in pure cultures of methanogens would be necessary to trace their exopolysaccharide production potential.

**Figure 7:**
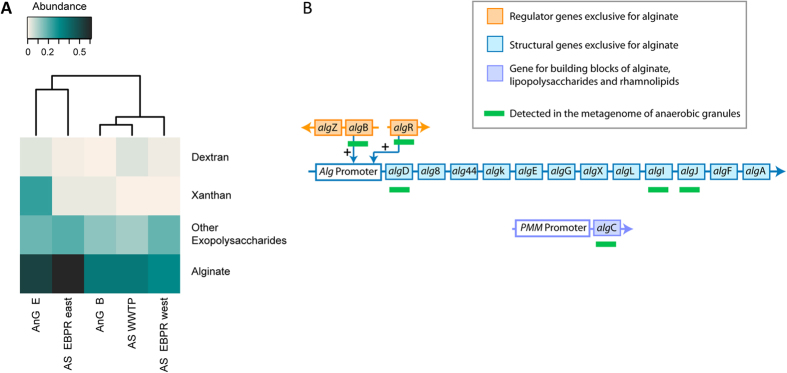
(**A**) Heat map showing the relative abundance of reads related to the polysaccharides dextran, xanthan, alginate and other polysaccharides in the metagenome of the anaerobic granules (AnG E and AnG B; note that the metagenome of AnG W was not available). Reads from metagenomes of activated sludge (AS), from two enhanced biological phosphorus removal (EBPR) plants, and from a conventional wastewater treatment plant (WWTP) are shown for comparison. (**B**) Simplified scheme of regulators and gene clusters of alginate biosynthesis showing the gene reads detected in the anaerobic granules. The positive sign indicates that those regulators initiate the transcription of the alginate operon. PMM refers to phosphomannomutase. Further details regarding the genetics of alginate biosynthesis can be found in references^45^,^46^.

Except for alginate, knowledge regarding genes involved in the production of microbial exopolysaccharides is limited. Thus, genetic approaches are restricted to the available databases. Nevertheless, the metagenome analysis of the granules showed reads related to exopolysaccharides metabolism (Fig. 7A). Dextran and xanthan reads were related to enzymes involved in the degradation of these polysaccharides; dextranases and xanthan lyases (Table S2), respectively. In contrast, alginate and other exopolysaccharides related reads were related to enzymes involved in their biosynthesis. Metagenomic data from various full-scale wastewater treatment systems showed the presence of alginate synthesis reads in flocculant activated sludge and also in anaerobic granules (Fig. 7A). Furthermore, alginate related reads were most abundant in both, the anaerobic granules and the flocculant activated sludge, compared to the other exopolysaccharides (Fig. 7A). In particular, sequence reads of the gene *alg*D for guanosine di-phospho (GDP)-mannose dehydrogenase (AlgD), which catalyzes the production of the key alginate precursor GDP-mannuronic acid^32^, were most abundant (Table S2, Fig. 7B). *alg*D is exclusive for the synthesis of alginate and is not known to be required for any other cellular process. Additionally, genes encoding alginate modifying enzymes *alg*I and *alg*J as well as regulatory genes, *alg*B and *alg*R, involved in initiating the transcription of the alginate operon were also found in the anaerobic granular sludges (Fig. 7B). The gene *alg*C was also detected; this gene is not alginate exclusive since it is required to produce building blocks of alginate, lipopolysaccharides and rhammnolipids. Based on MALDI-TOF spectra, AnG B and AnG W synthesized an alginate-like exopolysaccharide (Fig. 5) and the detection of sequence reads related to alginate synthesis in the metagome of the anaerobic granules thus linked genetic information with product formation.

Although reads related to xanthan degradation and to alginate synthesis were detected in AnG E, these polysaccharides could not be resolved from the MALDI-TOF spectra derived from these granules (Figure S4). It should be noted that metagenomic data only reflect the metabolic potential of the granules and do not provide information on the expression of certain genes. Therefore, future work should focus on the expression of genes involved in the production of microbial exopolysaccharides in anaerobic granules. However, these approaches are limited since, except for alginate, genes and associated proteins involved in the synthesis of exopolysaccharides in granular sludge remain unknown.

#### Implications

Though the presence of other exopolysaccharides which could not be detected by the techniques used in this study should not be excluded, findings based on the presence of (i) alginate-like exopolysaccharides in anaerobic granules treating different industrial wastewaters in this study, (ii) alginate-like exopolysaccharides in aerobic granules and in flocculant activated sludge in a previous study^33^, and (iii) reads related to the biosynthesis of alginate in the metagenomes of activated sludge from conventional and from enhanced biological phosphorus removal (EBPR) wastewater treatment plants and in the metagenomes of the anaerobic granules in the current study, suggest that alginate-like polysaccharide is a common exopolysaccharide produced by diverse microbial aggregates in biological wastewater treatment systems.

Collectively, the spectrometric and metagenomic results suggest that alginate-like exopolysaccharides can be constituents of the EPS matrix in anaerobic granular sludge. Using the same alkali-based extraction procedure, estimates of exopolysaccharides in anaerobic granules from different origins vary from 5 to 14 mg (mg VSS)^−1^ (this study, Table SI) and 10 to 50 mg (mg VSS)^−1^ 34. In aerobic granules, alginate-like polysaccharides can be key to maintaining the granular structure and the content of this polysaccharide can be high, ~160 mg (mg VSS)^−1^ 12. Although alginate-like polysaccharides may contribute to the granule structure in anaerobic granules from full-scale reactors as well, the inorganic precipitates found in their extracellular matrix^35^,^36^, which can even form skeleton-like structures as shown before for the AnG B granules^36^, may also contribute to the structure of these aggregates. The relative contribution of the various extracellular components to the granular structure or to granulation in full-scale reactors deserves further research particularly since various conditions from full-scale (i.e., industrial) systems cannot be simultaneously reproduced in laboratory systems, and most previous investigations in the determinants for granulation stem from laboratory-scale reactors.

Therefore, it is relevant to investigate extracellular components (e.g., EPS) in microbial aggregates from full-scale systems because those components are the matrix constituents of granules under real conditions. Particularly identifying those matrix constituents and their amounts from physically- and microbially- well functioning granules in relation to operational characteristics and microbial community composition of the reactors can help to envisage strategies to promote and maintain granular sludge under real full-scale conditions.

There has not been a detailed study on characterization of exopolysaccharides in anaerobic granules. This is mainly due to the difficulty in characterizing polysaccharides despite current advances in analytical tools. Although full chemical characterization of all exopolysaccharides of the anaerobic granules was not possible with the current tools, our approach of integrating their obtained spectra with multivariate analysis provided a basis to realizing that a common exopolysaccharide type can be produced in various anaerobic granular sludges. The finding of alginate-like exopolysaccharides in anaerobic granules expands the engineered environments where alginate has been found as EPS constituent. Our previous knowledge regarding alginate-like exopolysaccharide was based on aerobic granular sludges that were cultivated under anaerobic-aerobic conditions^12^, and where the anaerobic phase represents a significant fraction of the operation time. The environmental trait(s) triggering alginate-like synthesis are not known, however, it is possible that anaerobic, and not aerobic conditions trigger alginate-like production in aerobic granules. This is supported by Bragonzi *et al.* (2005)^37^ who found that anaerobic conditions increased bacterial alginate production in medical relevant biofilms. Thus, it warrants investigating further to what extent anaerobic environments trigger the synthesis of this polysaccharide in wastewater treatment biofilms. Future improvements in analytical tools could furthermore help in elucidating the detailed chemical structure of exopolysacharides and the genes responsible for their production in complex microbial communities.

Considering the relevance of exopolysaccharides in wastewater treatment systems and given that characterizing exopolysaccharides from mixed microbial systems is not straightforward, it is proposed that the use of ^1^H NMR, and/or MALDI-TOF, combined with NMDS could potentially be used as a method for monitoring or comparing profiles of exopolysaccharides from various microbial aggregates such as granules and flocs as well as biofilms in biological wastewater treatment systems and as a function of operational conditions.

## Methods

### Granular Sludge

All granular sludges were collected from full-scale reactors in The Netherlands. The anaerobic granules were from upflow anaerobic reactors treating paper mill (AnG E), brewery (AnG B) and sulfate-rich thermophilic (AnG W) wastewaters. Information regarding operational conditions of the papermill and the brewery reactors have been previously reported^36^,^38^, whereas information of the third reactor could not be retrieved from the company.

### Extraction of EPS

Two methods were used to extract (crude) EPS including the cation exchange resin (CER) method^39^ and the formaldehyde-NaOH procedure^6^ as described by Seviour *et al.*^40^ with some modifications (see the Supporting Information). Since preliminary tests showed that the formaldehyde-NaOH procedure resulted in higher EPS yields (1.6–2 fold) compared to the CER method, this method was used for the remainder of the experiments. Negligible contamination due to cell lysis during extraction was verified as specified in Table S1. The protein fraction of EPS was precipitated using 70% (v/v) perchloric acid to a 5% (v/v) final concentration^40^. After incubation at 4 °C for 5 min, the sample was centrifuged at 12,000 × g for 20 min at 4 °C. The polysaccharide fraction was recovered from the supernatant and was further neutralized and dialyzed against ultrapure water for 48 h^40^. Polysaccharides were then precipitated in cold absolute ethanol to a 80% (v/v) final concentration^12^ and incubated at 4 °C for a minimum of 24 h. After centrifugation at 12,000 × g for 20 min at 4 °C, ethanol was removed and the polysaccharide fraction was lyophilized. The content of proteins and carbohydrates in EPS was determined as described previously^39^.

### Nuclear Magnetic Resonance (NMR) Spectroscopy

Samples of reference polysaccharides were prepared by dissolving separately 8 mg of alginate (Sigma Aldrich, CAS No. 9005-32-7), 8 mg of gellan (AppliChem, CAS No. 71010-52-1) or 3 mg of xanthan (Sigma Aldrich, CAS No.11138-66-2) in 1 mL of 0.1M NaOH in deuterium oxide (D2O, 99.9%)^10^. Higher xanthan concentrations were not used as they were not soluble. Polysaccharide samples from the granules were prepared in a similar manner except that 1.5 to 3 mg of lyophilized polysaccharide fraction was used. The NMR spectra were acquired at 330K using a 950 AVANAC III spectrometer equipped with a CP TCI multinuclear CryoProbe (BrukerBioSpin, Rheinstetten, Germany). To achieve an optimum signal-to-noise ratio, the ^1^H NMR spectra were recorded by collecting 1k scans with a recycle delay time of 3 sec. To suppress the water peak, each spectrum was induced with an excitation sculpting pulse sequence using the standard (zgespg) program from the Bruker pulse library. Chemical shifts were determined relative to 3-trimethylsilylpropane sulfonic acid (DSS, 20 μM) as internal chemical shift reference. The free induction decay (FID) data were collected with spectral width of 15243 Hz digitized into 64k data points. The FID signals were zero-filled and amplified by an exponential line-broadening factor of 0.3 Hz before Fourier transformation. The Topspin 2.1 software (Bruker) was used in all experiments to collect and analyze data.

The similarities between NMR spectra were visually represented using non-metric multidimensional scaling (NMDS). Compared to other ordination methods, NMDS does not assume linear relationships or normal distributions of data. The algorithm iteratively searches to place samples as points on k dimensions (axis) while attempting to maintain the relative distances of points as close as possible to the actual rank order of similarities between samples. Thus, samples having similar NMR spectra are plotted together in the ordination space.

The polysaccharide regions (3.2 to 5.6 ppm) of the NMR spectra were subdivided into sequential 0.2 ppm regions; each region was integrated (using the Topspin 2.1 software) and normalized (normalization accounts for differences in amount of sample used) in proportion to the total area of the polysaccharide region. With this data set, NMDS analysis and hierarchical clustering with the complete linkage method was carried out using R Studio (http://www.rstudio.org/) and the community ecology package vegan (http://CRAN.R-project.org/package=vegan) using the Bray-Curtis coefficient for calculating the dissimilarity matrix.

### Matrix-Assisted Laser Desorption/Ionization Time-Of-Flight Mass Spectrometry (MALDI-TOF MS)

Samples were mixed at a 1:1 ratio with 2,5-dihydroxybenzoic acid (DHB). The DHB matrix was prepared at 20 mg mL^−1^ concentration and dissolved in TA30 (30% [v/v] acetonitrile, 0.1% [v/v] trifluoroacetic acid in distilled water). A 2 μL volume of the sample-matrix mixture was spotted on a MTP Anchorchip 600–384 (Bruker Daltonic Gmbh, Bremen, Germany) target plate. Similar to the samples, the reference polysaccharides dextran, gellan, xanthan and alginate (3 mg mL^−1^) were prepared for analysis. The DHB matrix has been previously used for the analysis of oligo and polysaccharides^21^. Mass spectra were obtained with an Ultraflex III matrix assisted laser desorption ionization-time-of-flight/time-of-flight (MALDI-TOF/TOF) tandem mass spectrometer (MS/MS; Bruker Daltonics) equipped with a nitrogen laser emitting at 337 nm, a 3 ns pulse width, and operated in linear positive mode with the flexControl software (version 3.3.108.0; Bruker Daltonics) and a reflector voltage of 26.3 kV. The laser was used at a repetition rate of 200 Hz and power was manually adjusted until obtaining optimum signal-to-noise ratio, generally comprising between 40–60%. Each acquired spectrum resulted from the accumulation of a minimum of 4000 laser shots.

### 16S rRNA Gene and Metagenome Pyrosequencing

Genomic DNA was extracted from the granules using the Power Soil DNA isolation kit (MoBio, Inc.); see SI. High-throughput pyrosequencing of 16S rRNA gene fragments and quantitative-PCR of bacteria and archaea was conducted as detailed in the Supporting Information. The raw 16S rRNA pyrosequencing reads were deposited in the NCBI Short-Read Archive under BioProject number PRJNA242588. Specifically, data sets of the sequence reads for the different granular sludge can be found under the following NCBI BioSample accession numbers: SAMN02649632 for the sulfate-rich thermophilic granules, SAMN02649633 for the brewery granules, and SAMN02649634 for the paper mill granules.

Shotgun metagenome sequencing of the granules was conducted as described in the Supporting Information. After removing low quality reads and ambiguous bases, unassembled sequences were uploaded into the MG-RAST server for annotation^41^. The MG-RAST accession numbers of the granules are as follows: AnG B (4559623.3) and AnG E (4547780.3). The metagenome of the AnG W could not be analyzed due to sample limitations. The abundance of sequence reads related to exopolysaccharides metabolism (or synthesis/degradation) in the metagenomes of the anaerobic granules was compared to those found in the metagenomes of three full-scale activated sludge wastewater treatment systems available on MG-RAST (Table S2).

## Additional Information

**How to cite this article**: Gonzalez-Gil, G. *et al.* NMR and MALDI-TOF MS based characterization of exopolysaccharides in anaerobic microbial aggregates from full-scale reactors. *Sci. Rep.* **5**, 14316; doi: 10.1038/srep14316 (2015).

## Supplementary Material

Supplementary Information
