# Supplementary material for: NMR and MALDI-TOF MS based characterization of exopolysaccharides in anaerobic microbial aggregates from full-scale reactors
Source: Sci Rep. 2015 Sep 22;5:14316. doi: 10.1038/srep14316 (PMC4585762; doi:10.1038/srep14316)
Supplement: Supplementary Information [file srep14316-s1.pdf]

Supporting Information

NMR and MALDI-TOF MS based characterization of exopolysaccharides in anaerobic microbial aggregates from full-scale reactors

Graciela Gonzalez-Gil [1,2*], Ludivine Thomas[3] , Abdul-Hamid Emwas[4], Piet N.L. Lens[2] , Pascal E. Saikaly[1]

[1] King Abdullah University of Science and Technology, Water Desalination and Reuse Center. Thuwal, Saudi Arabia.

[2] UNESCO-IHE, Westvest 7, 2611 AX Delft, the Netherlands.

[3] King Abdullah University of Science and Technology, Biosciences Core Laboratory, Thuwal, Saudi Arabia.

[4]King Abdullah University of Science and Technology, Advanced Nanofabrication Imaging and Characterization, Thuwal, Saudi Arabia.

*Corresponding author: g.gonzalezgil@unesco-ihe.org ; graciella.gil@gmail.com

**Corresponding author: pascal.saikay.@kaust.edu.sa

Supporting methods

Extraction of crude EPS

Granular sludge was briefly washed twice in phosphate buffer saline (pH 7) to remove non-granular material. Then, 10 mL of 0.1 M NaCl was added to 3-5 g of granular sludge and the granules were mechanically disrupted using a pestle to obtain a slurry. Immediately after adding 10 mL of water, formaldehyde was added to a final concentration of 0.4% (v/v) and the slurry was incubated at 4°C for 1 h. Then, 10 mL of 0.1 M NaOH was added and the slurry was incubated at 4°C for 3 h. The sample was then centrifuged (12,000 × *g*, 20 min at 4°C) and the supernatant filtered (0.45 μm) to recover the (crude) extracted EPS. This EPS solution was neutralized with 1M HCl.

DNA extraction from the granules

Prior to DNA extraction, a slurry of the granules was prepared by mechanical disruption in PBS buffer. Genomic DNA was extracted, in duplicate, from 0.3 to 0.4 ml of slurry using the Power Soil DNA isolation kit (MoBio, Inc.) according to the manufacturer's protocol except that two cycles of bead beating for 30 s (each cycle followed by 30 s on ice) were used to improve cell lysis. The duplicate DNA extracts were then pooled and the DNA concentration was measured using a NanoDrop-1000 spectrophotometer (Thermo Scientific, Waltham, MA).

Quantitative PCR

Quantitative PCR was conducted in triplicate using a CFX96 real time detection apparatus (Bio-Rad Laboratories, Hercules, CA). Each 25 μl reaction contained 12.5 μl of iQ SYBR Green Super Mix (Bio-Rad Laboratories, Hercules, CA), 300 nM of each forward and reverse bacterial or archaeal primers as defined below and 2 μl of template DNA. Cycling conditions

were: 3 min at 95°C, 40 cycles of 30 s at 94°C, 30 s at 56°C (53 °C for archaea) and 30 s at 72°C. After cycling, a melting curve analysis from 50 to 95°C was conducted to verify the specificity of the reactions. To quantify copy numbers of bacterial and archaeal 16S rRNA genes, standard curves for bacteria and archaea were constructed for each qPCR run by using dilution series of known concentrations of purified bacteria and archaea amplicons amplified using the same primer sets (described below) and genomic DNA from *Geobacter sulfurreducens* and *Methanosarcina barkeri*, respectively.

DNA amplification and 454 pyrosequencing of 16S rRNA gene amplicons

Bacterial and archaeal 16S rRNA gene fragments were amplified using primers B-341F (5'-CCTACGGGNGGCWGCAG -3') and B-805R (5'-GACTACHVGGGTATCTAATCC -3') for *Bacteria*, and A-519F (5'-CAGCMGCCGCGGTAA -3') and A-1017R (5'-GGCCATGCACCWCCTCTC-3') for *Archaea*. These primer combinations were selected as they result in high domain coverage and specificity based on a recent study.[1] Extracted DNA was amplified in triplicates using platinum PCR SuperMix High Fidelity Taq DNA Polymerase reagent, primer concentrations were 300 nM each and DNA template was 20 to 40 ng. The thermocycling consisted of an initial step at 94°C for 2 min, followed by 25 cycles at 94°C for 30 s, 56°C (53°C for archaea) for 40 s, and 72°C for 60 s, with a final extension at 72°C for 7 min. Products were checked for correct length by gel electrophoresis.

For pyrosequencing, a second round of PCR [2] was performed for which the 454-adaptor sequence A (5'-ccatctcatccctgcgtgtctccgactcag- 3') followed by a sample specific 8 nt barcode sequence were attached to the 5' end of the forward primers, while the 454-adaptor sequence B (5'-cctatcccctgtgtgccttggcagtctcag-3') was attached to the 5' end of the reverse primers. The barcodes we used[3] met the following criteria[4]: (i) differed by at least 3 nt, (ii) did not contain homopolymers, (iii) the end nt of the adapter differed from the start nt of the

barcode, (iv) the end nt of the barcode differed from the start nt of the primer and (v) barcodes had a similar GC content. The 50 μl PCR mixture contained 45 μl of Platinum PCR SuperMix High Fidelity *Taq* DNA Polymerase reagent (Invitrogen, Carlsbad, CA) and 500 nM of forward primer, 300 nM of reverse primer and 2 μl of template. The thermocycling consisted of an initial step at 94°C for 2 min, followed by 12 cycles at 94°C for 30 s, 56°C for 40 s, and 72°C for 60 s, with a final extension at 72°C for 7 min. Products of triplicate reactions were pooled for each granule sample and then purified using the QIAquick kit PCR purification kit (Qiagen, Valencia, CA) according to the manufacturer's protocol and amplicon concentrations were quantified (NanoDrop-1000 spectrophotometer, Thermo Scientific, Waltham, MA). Then, amplicons from different granule samples were mixed to obtain equal mass concentrations in the final mixture. This mixture was further gel-purified using the QIAquick Gel extraction kit (Qiagen, Valencia, CA) according to the manufacturer's protocol.

The 16S rRNA gene fragments were pyrosequenced at the Biosciences Core Laboratory at KAUST using a Roche 454 GS FLX sequencer and Titanium reagents according to the manufacturer's protocols.

Analysis of 16S rRNA gene pyrosequencing data

Sequence analyses were conducted using the Quantitative Insights Into Microbial Ecology (QIIME) pipeline (http://qiime.org) as described by Caporaso et al.[5] Briefly, sequences were quality filtered by eliminating reads that were shorter than 200 bp, had a quality score lower than 25, and did not perfectly match the PCR primer and the barcode sequence. After denoising, sequences were clustered into operational taxonomic units (OTUs) at ≥97% sequence similarity using Uclust[6] as implemented in QIIME. After selecting a representative sequence for each OTU, sequences were aligned using PyNAST[7] and their taxonomic identity

was assigned using the RDP classifier (Wang et al. 2007) with the Greengenes (v 10-12) as reference database. Chimeric sequences were detected using ChimeraSlayer[8] and removed from further analysis. Then, a phylogenetic tree was constructed using the chimeric-free representative sequences and a table of OTU counts per sample was generated.

Metagenomic analysis

DNA extracted as specified above was sequenced using the Roche GS-FLX titanium sequencer. After removing low quality reads and ambiguous bases, unassembled sequences were uploaded into the MG-RAST server for annotation[9]. The annotation of reads was done using the IMG database of MG-RAST, using the default parameters i.e., cutoff expectation value (E) of 10^{-5}, a minimum alignment length of 15 aminoacids or base pairs and a minimum identity cutoff of 60%.

Supporting figures and tables

Table S1. Protein (P), carbohydrate (C) and extracellular DNA (eDNA) contents in the extracellular polymeric substances extracted from various types of granular sludges[#].

Granules	mg $(gVSS)^{-1}$			P/C
	**Protein**	**Carbohydrate**	**eDNA**	
AnG B	11.0±3.4	13.5±3.5	0.08±0.04	0.8
AnG W	2.4±0.4	12.3±2.2	n.a.	0.2
AnG E	4.3±0.7	5.1±0.3	0.05±0.02	0.9

A proteomic analysis as described by Thomas et al.[10] of the EPS extracts, did not show the presence of the cytosolic enzyme glucose-6–phosphate-dehydrogenase The presence of this enzyme in EPS extracts has been associated to a high extent of cell lysis during the extraction procedure.[11] The absence of this enzyme thus suggests that limited or no cell lysis occurred during the EPS extraction from the granular sludges. Also P/C ratios < 2 and low contents of extracellular DNA (e.g. < 0.6 mg DNA $(gVSS)^{-1}$), as measured in this study, exclude significant occurrence of cell rupture during EPS extraction.[12,13]

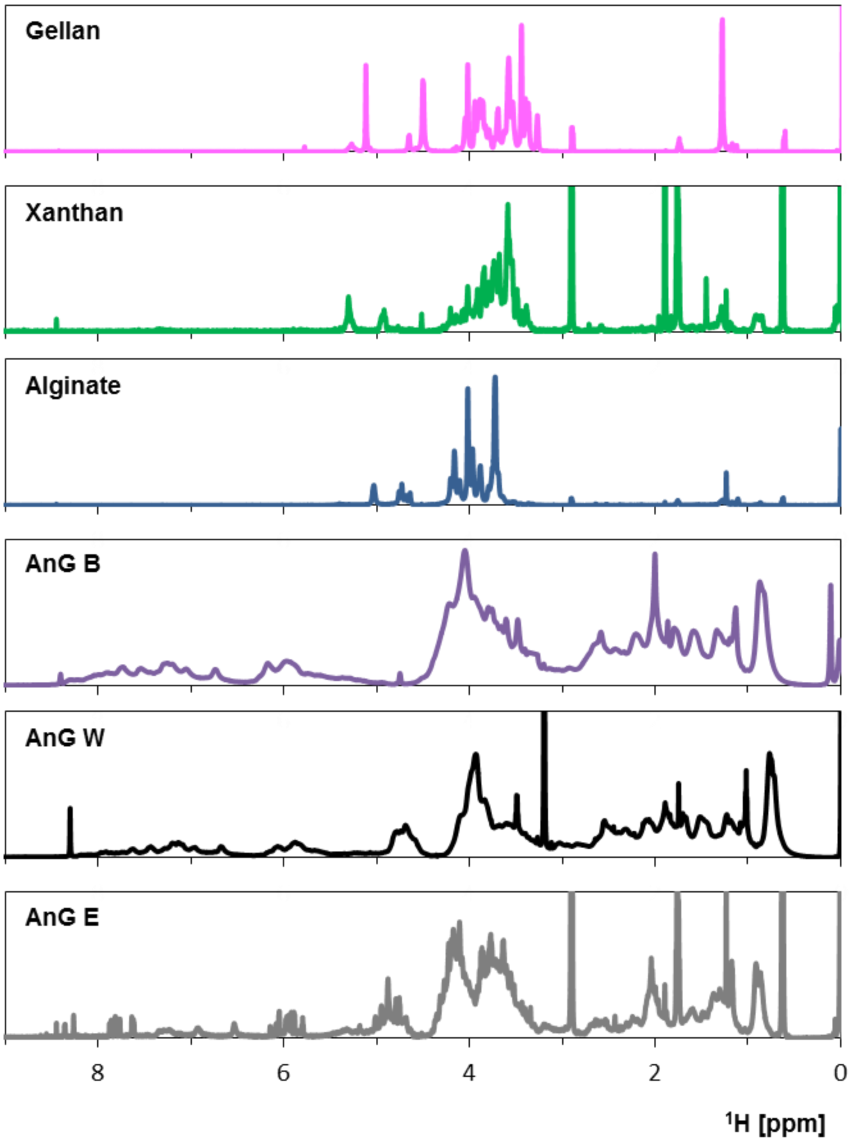

Figure S1. ^{1}H NMR spectra of reference polysaccharides and from exopolysaccharides extracted from anaerobic granules.

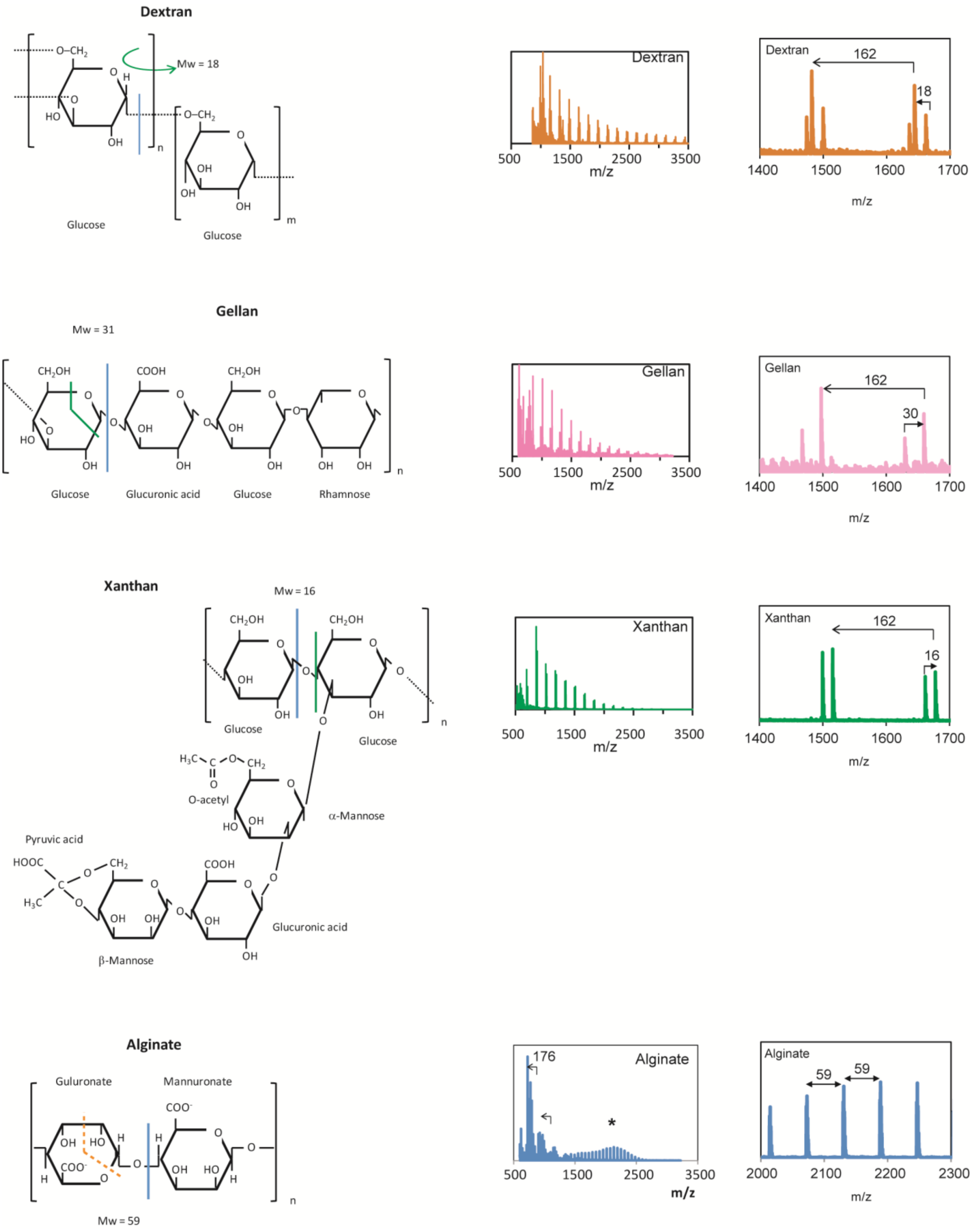

Figure S2. Chemical structure of reference polysaccharides. The blue line indicates the proposed fragmentation sites in the glycosidic bonds during MALDI-TOF MS analysis [14]. The green lines indicate the proposed fragmentation resulting in the satellite ion peaks as observed in the MALDI-TOF MS spectra; the corresponding mass difference (as Mw) is given. For dextran, a process involving loss of water is proposed. Details on ion formation and fragmentation aspects in mass spectrometry can be found in reference [15]. For alginate, the orange dashed line indicates a proposed cross-ring breakage that could result in ions having peak differences of 59 m/z.

Multivariate analysis of MALDI-TOF MS spectra

The MALDI-TOF MS spectra peak list data was used for analysis. For the alginate and granules samples the following manual filter was used due to the noisy signals from 600 to 1600 m/z range. In this region, and for these samples, only the peaks with the highest intensities were retained. From the rest of the m/z range, all peaks were retained. Spectra were normalized related to the maximum peak of each spectrum. However, in further studies, more refined and automated processing of raw spectra should be investigated. Other studies have demonstrated the use of MALDI-TOF MS combined with multivariate analysis (e.g., MDS) to identify bacterial species[16], characterize the proteolysis of cheese [17] and keratin in furs [18].

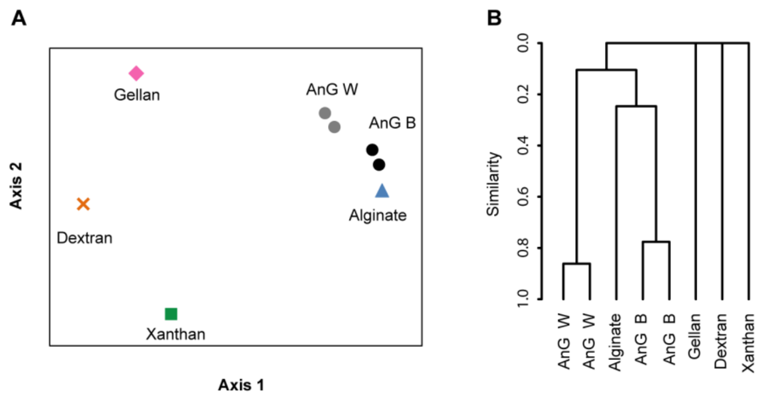

Figure S3. A) Similarities between the MALDI-TOF MS spectra of reference polysaccharides and polysaccharides extracted from various granular sludges represented by non-parametric multidimensional scaling. Stress = 0.06. B) Hierarchical clustering of reference and granular sludge polysaccharides based on Bray-Curtis similarity.

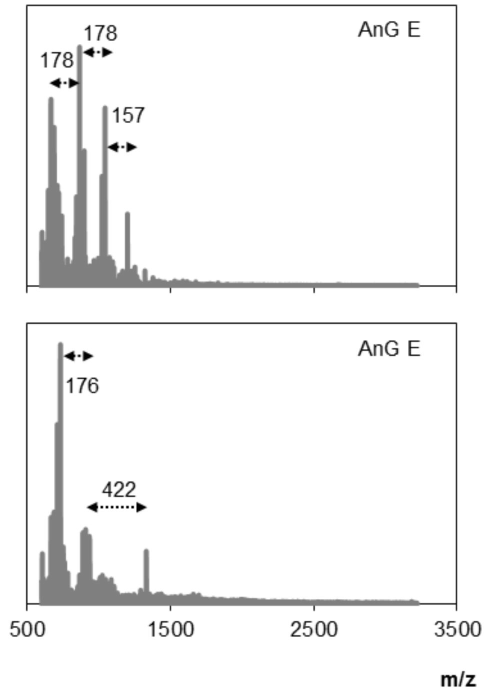

Figure S4. MALDI-TOF MS spectra of exopolysaccharides from anaerobic granules AnG E. The matrix used was 2,5-dihydroxybenzoic acid (DHB) and spectra were acquired in the positive ion mode.

Microbial community of the anaerobic granules

Bacteria belonging to the phylum *Proteobacteria*, and to the classes *Clostridia* and *Bacteroidia* are known to produce exopolysaccharides.[19-21] All these bacterial types were present in the granular sludges (Figure S5a). δ-*Proteobacteria* (33%) and specifically the family *Geobacteracea* (17%) were most abundant in the AnG B granules. The identity of exopolysaccharides material produced by this family is not known. It was postulated that *Geobacter* species can directly (i.e., cell-to-cell) transfer electrons to methanogens during the anaerobic conversion of ethanol.[22], which may explain its high abundance in these granules treating brewery wastewater, although this physiological trait warrants further investigation. Regarding the paper-mill AnG E granules, *Bacteroidia* (29%) and *Clostridia* (16%) were among the main bacterial types. The first bacterial class may degrade polysaccharides and other complex substrates to sugars[23], while the latter may be involved in fermentation reactions[24] and their abundance may correspond with starch being the main carbohydrate in the wastewater of the paper-mill reactor.[25] Conversely, the main bacteria in the thermophilic AnG W granules were closely affiliated to clones within *Thermodesulfovibrio* (Class *Nitrospira*, Figure S5a), which are thermophilic sulfate reducing bacteria.[26]

The abundance of archaea in the anaerobic granules, based on quantitative-PCR, was about 10 to 14% of the microbial community. Most of the methanogens were affiliated to the classes *Methanobacteria* and *Methanomicrobia* which comprise families of hydrogenotrophic and acetoclastic methanogens, respectively.

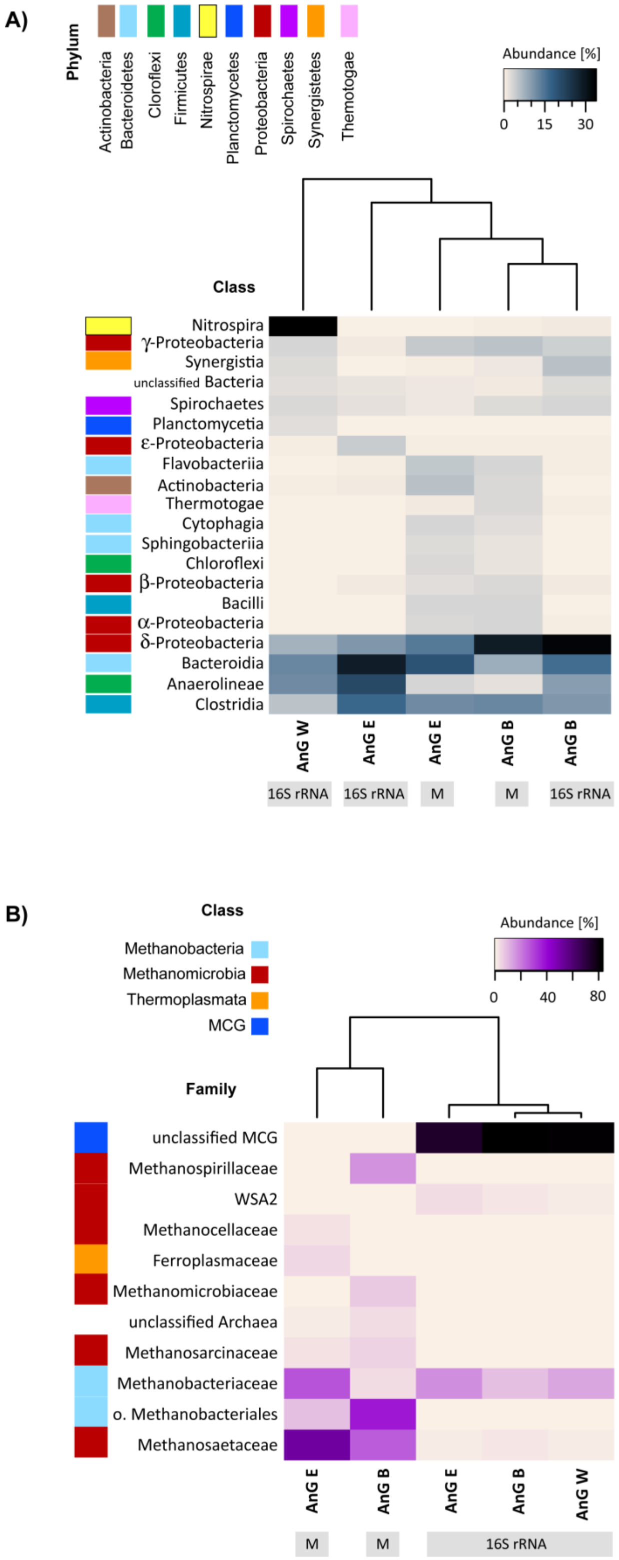

Figure S5. Heat maps showing the (A) bacterial and (B) archaeal community structure of the anaerobic granules (AnG) obtained by pyrosequencing analysis of 16S rRNA gene fragments (16S rRNA) or by metagenomic analysis (M). Bacteria and archaea with abundance < 2% are not plotted. Dominant bacteria reflected by the 16S rRNA and the metagenomic analysis correspond well except for some under-representation of *Anaerolinea* in the metagenomic data set. In the archaeal community, *Methanobacteriacea* corresponds well in the 16S rRNA and the methagenome data sets. However, the 16S rRNA pyrosequencing data set appeared to under amplify *Methanomicrobia* while apparently over amplifying the Crenarchaeota MCG group.

Table S2. Exopolysaccharides associated reads from metagenomic data. Parameters used were: IMG database, maximum e-value cutoff=1e-5, minimum % identity cutoff=60%, and min. alignment length cutoff=15. The EBPR sludge was taken from the East and West wastewater treatment plants of the city of Aalborg, Denmark, and details regarding sludge sampling and processing can be found in reference (27).

**MG-RAST ID:**<br>**Sample:**	4559623<br>AnG B	4547780<br>AnG E	4463936<br>AS EBPR East	4455295<br>AS EBPR West	4467420<br>AS WWTP
**Alginate related reads:**					
AlgC					2
AlgI	1	3			
AlgI2		1			
Alginate biosynthesis protein AlgD				1	1
Alginate biosynthesis regulatory protein AlgR				1	
Alginate biosynthesis sensor protein KinB (EC 2.7.3.-)			2		
Alginate lyase AlgL					1
Alginate O-acetylation protein	4	11	10	2	2
Alginate O-acetylation protein / membrane bound O-acyl transferase, MBOAT			1		
Alginate O-acetylation protein AlgI			3	1	
Alginate O-acetylation protein, putative alginate o-acetyltransferase	1		3		
Alginate O-acetyltransferase			4		
Alginate O-acetyltransferase AlgI			3	3	2
Alginate O-acetyltransferase AlgJ		1			
Alginate regulatory hybrid histidine protein kinase GacS			1		
GDP-mannose dehydrogenase	21	22	35	16	5
GDP-mannose 6-dehydrogenase (algD)		6			
Oligosaccharide biosynthesis protein Alg14 like protein				1	
Phosphomannomutase AlgC		1			
Possible phosphomannomutase AlgC					10
Putative alginate biosynthesis protein AlgI			2		
Putative alginate lyase protein					1
Putative alginate O-acetyltransferase			1	3	10
Putative alginate O-acetyltransferase AlgI		2		1	
Putative exported serine protease, HtrA/AlgW-like protein			1		5
Putative poly(beta-D-mannuronate) O-acetylase (Alginate biosynthesis protein algI)		1			
Putative transcriptional activator of alginate synthesis	1				
Regulator of RpoD, Rsd/AlgQ					3
Response regulator of the LytR/AlgR family	4	9	12	1	
RNA polymerase sigma factor AlgU			1		
Sensory transduction protein kinase AlgZ (EC 2.7.3.-)				2	
Transcriptional regulator, LytR/AlgR family			6	16	
Two-component response regulator AlgB	3	5	2		
**Dextran related reads:**					
Dextransucrase		2			
Dextranase (EC:3.2.1.11)		2			
Glucodextranase		1		2	6
Alpha-dextran endo-1,6-alpha-glucosidase			1	1	
Putative dextran glucosidase			1	1	2

MG-RAST ID:	4559623	4547780	4463936	4455295	4467420
Sample:	AnG B	AnG E	AS EBPR East	AS EBPR West	AS WWTP
**Xanthan related reads:**					
Exported xanthan lyase/N-acetylmuramoyl-L-alanine amidase	1	1	2		
Probable secreted protein-putative xanthan lyase related	1	5			
Probable xanthan lyase		6	1	1	
Putative exported xanthan lyase/N-acetylmuramoyl-L-alanine amidase		4			
Putative xanthan lyase XalB precursor		2			
Secreted protein-putative xanthan lyase related		5			
Xanthan lyase		13			
Xanthan biosynthesis chain length determinant protein GumC					
**Exopolysaccharides related reads:**					
Capsular exopolysaccharide family	5	10	17	10	
Exopolysaccharide biosynthesis acetyltransferase					1
Exopolysaccharide biosynthesis polyprenyl glycosylphosphotransferase	8	8	17	19	1
Exopolysaccharide biosynthesis protein		1	1	5	
Exopolysaccharide biosynthesis protein related to N-acetylglucosamine-1-phosphodiester Alpha-N-acetylglucosaminidase		1	1	1	
Exopolysaccharide biosynthesis protein-like				2	
Exopolysaccharide production CDP-diacylglycerol-serine O-phosphatidyltransferase protein			1		
Exopolysaccharide production negative regulator ExoR					2
Exopolysaccharide production negative regulator precursor					2
Exopolysaccharide production protein ExoQ		1			
Exopolysaccharide production protein ExoY					3
Exopolysaccharide synthesis, ExoD		1		3	
Exopolysaccharide transport protein					
Exopolysaccharide transport protein, putative			1		
Exopolysaccharide xanthan biosynthesis chain length determinant protein GumC			1		
Exopolysaccharide/PEPCTERM locus tyrosine autokinase				1	
Putative exopolysaccharide biosynthesis protein		3	3	3	
Putative exopolysaccharide biosynthesis protein (protein-tyrosine kinase)		1			
Putative exopolysaccharide production negative regulator precursor					2
Putative exopolysaccharide production protein (ExoQ-like); putative membrane protein					3
Putative exopolysaccharide regulatory protein exoR					4
Putative membrane protein of ExoQ family involved in exopolysaccharide production					1

Supporting references

(1) Klindworth, A.; Pruesse, E.; Schweer, T.; Peplies, J.; Quast, C.; Horn, M.; Glöckner, F. O., Evaluation of general 16S ribosomal RNA gene PCR primers for classical and next-generation sequencing-based diversity studies. *Nucleic Acids Research* **2013,** *41*, (1), e1.

(2) Behrendt, L.; Larkum, A. W. D.; Trampe, E.; Norman, A.; Sorensen, S. J.; Kuhl, M., Microbial diversity of biofilm communities in microniches associated with the didemnid ascidian Lissoclinum patella. *ISME J* **2012,** *6*, (6), 1222-1237.

(3) Meyer, M.; Stenzel, U.; Hofreiter, M., Parallel tagged sequencing on the 454 platform. *Nat. Protocols* **2008,** *3*, (2), 267-278.

(4) Parameswaran, P.; Jalili, R.; Tao, L.; Shokralla, S.; Gharizadeh, B.; Ronaghi, M.; Fire, A. Z., A pyrosequencing-tailored nucleotide barcode design unveils opportunities for large-scale sample multiplexing. *Nucleic Acids Research* **2007,** *35*, (19), e130.

(5) Caporaso, J. G.; Kuczynski, J.; Stombaugh, J.; Bittinger, K.; Bushman, F. D.; Costello, E. K.; Fierer, N.; Pena, A. G.; Goodrich, J. K.; Gordon, J. I.; Huttley, G. A.; Kelley, S. T.; Knights, D.; Koenig, J. E.; Ley, R. E.; Lozupone, C. A.; McDonald, D.; Muegge, B. D.; Pirrung, M.; Reeder, J.; Sevinsky, J. R.; Turnbaugh, P. J.; Walters, W. A.; Widmann, J.; Yatsunenko, T.; Zaneveld, J.; Knight, R., QIIME allows analysis of high-throughput community sequencing data. *Nat Meth* **2010,** *7*, (5), 335-336.

(6) Edgar, R. C., Search and clustering orders of magnitude faster than BLAST. *Bioinformatics* **2010,** *26*, 2460-2461.

(7) Caporaso, J. G.; Bittinger, K.; Bushman, F. D.; DeSantis, T. Z.; Andersen, G. L.; Knight, R., PyNAST: a flexible tool for aligning sequences to a template alignment. *Bioinformatics* **2010,** *26*, 266-267.

(8) Haas, B. J.; Gevers, D.; Earl, A. M.; Feldgarden, M.; Ward, D. V.; Giannoukos, G.; Ciulla, D.; Tabbaa, D.; Highlander, S. K.; Sodergren, E.; Methé, B.; DeSantis, T. Z.; Consortium, T. H. M.; Petrosino, J. F.; Knight, R.; Birren, B. W., Chimeric 16S rRNA sequence formation and detection in Sanger and 454-pyrosequenced PCR amplicons. *Genome Research* **2011,** *21*, (3), 494-504.

(9) Meyer, F.; Paarmann, D.; D'Souza, M.; Olson, R.; Glass, E.; Kubal, M.; Paczian, T.; Rodriguez, A.; Stevens, R.; Wilke, A.; Wilkening, J.; Edwards, R., The metagenomics RAST server - a public resource for the automatic phylogenetic and functional analysis of metagenomes. *BMC Bioinformatics* **2008,** *9*, (1), 386.

(10) Thomas, L.; Marondedze, C.; Ederli, L.; Pasqualini, S.; Gehring, C., Proteomic signatures implicate cAMP in light and temperature responses in Arabidopsis thaliana. *Journal of Proteomics* **2013,** *83*, (0), 47-59.

(11) Frølund, B.; Palmgren, R.; Keiding, K.; Nielsen, P. H., Extraction of extracellular polymers from activated sludge using a cation exchange resin. *Water Research* **1996,** *30*, (8), 1749-1758.

(12) Comte, S.; Guibaud, G.; Baudu, M., Relations between extraction protocols for activated sludge extracellular polymeric substances (EPS) and EPS complexation properties: Part I. Comparison of the efficiency of eight EPS extraction methods. *Enzyme and Microbial Technology* **2006,** *38*, (1–2), 237-245.

(13) Adav, S. S.; Lee, D. J., Extraction of extracellular polymeric substances from aerobic granule with compact interior structure. *Journal of Hazardous Materials* **2008,** *154*, (1-3), 1120-1126.

(14) Harvey, D. J., Matrix-assisted laser desorption/ionization mass spectrometry of carbohydrates. *Mass Spectrometry Reviews* **1999,** *18*, (6), 349-450.

(15) Domon, B.; Costello, C., A systematic nomenclature for carbohydrate fragmentations in FAB-MS/MS spectra of glycoconjugates. *Glycoconj J* **1988,** *5*, (4), 397-409.

(16) De Bruyne, K.; Slabbinck, B.; Waegeman, W.; Vauterin, P.; De Baets, B.; Vandamme, P., Bacterial species identification from MALDI-TOF mass spectra through data analysis and machine learning. *Systematic and Applied Microbiology* **2011,** *34*, (1), 20-29.

(17) Piraino, P.; Upadhyay, V. K.; Rossano, R.; Riccio, P.; Parente, E.; Kelly, A. L.; McSweeney, P. L. H., Use of mass spectrometry to characterize proteolysis in cheese. *Food Chemistry* **2007,** *101*, (3), 964-972.

(18) Hollemeyer, K.; Altmeyer, W.; Heinzle, E.; Pitra, C., Matrix-assisted laser desorption/ionization time-of-flight mass spectrometry combined with multidimensional scaling, binary hierarchical cluster tree and selected diagnostic masses improves species identification of Neolithic keratin sequences from furs of the Tyrolean Iceman Oetzi. *Rapid Communications in Mass Spectrometry* **2012,** *26*, (16), 1735-1745.

(19) Vu, B.; Chen, M.; Crawford, R. J.; Ivanova, E. P., Bacterial extracellular polysaccharides involved in biofilm formation. *Molecules* **2009,** *14*, (7), 2535-2554.

(20) Giallo, J.; Gaudin, C.; Belaich, J. P.; Petitdemange, E.; Caillet-Mangin, F., Metabolism of glucose and cellobiose by cellulolytic mesophilic Clostridium sp. strain H10. *Applied and Environmental Microbiology* **1983,** *45*, (3), 843-849.

(21) Braissant, O.; Decho, A. W.; Dupraz, C.; Glunk, C.; Przekop, K. M.; Visscher, P. T., Exopolymeric substances of sulfate-reducing bacteria: Interactions with calcium at alkaline pH and implication for formation of carbonate minerals. *Geobiology* **2007,** *5*, (4), 401-411.
(22) Morita, M.; Malvankar, N. S.; Franks, A. E.; Summers, Z. M.; Giloteaux, L.; Rotaru, A. E.; Rotaru, C.; Lovley, D. R., Potential for direct interspecies electron transfer in methanogenic wastewater digester aggregates. *mBio* **2011,** *2*, (4).
(23) Xu, J.; Bjursell, M. K.; Himrod, J.; Deng, S.; Carmichael, L. K.; Chiang, H. C.; Hooper, L. V.; Gordon, J. I., A genomic view of the human-bacteroides thetaiotaomicron symbiosis. *Science* **2003,** *299*, (5615), 2074-2076.
(24) Lin, C.-Y.; Lay, C.-H.; Sen, B.; Chu, C.-Y.; Kumar, G.; Chen, C.-C.; Chang, J.-S., Fermentative hydrogen production from wastewaters: A review and prognosis. *International Journal of Hydrogen Energy* **2012,** *37*, (20), 15632-15642.
(25) Janssen, A. J. H.; Lens, P. N. L.; Stams, A. J. M.; Plugge, C. M.; Sorokin, D. Y.; Muyzer, G.; Dijkman, H.; Van Zessen, E.; Luimes, P.; Buisman, C. J. N., Application of bacteria involved in the biological sulfur cycle for paper mill effluent purification. *Science of the Total Environment* **2009,** *407*, (4), 1333-1343.
(26) Sekiguchi, Y.; Muramatsu, M.; Imachi, H.; Narihiro, T.; Ohashi, A.; Harada, H.; Hanada, S.; Kamagata, Y., *Thermodesulfovibrio aggregans* sp. nov. and *Thermodesulfovibrio thiophilus* sp. nov., anaerobic, thermophilic, sulfate-reducing bacteria isolated from thermophilic methanogenic sludge, and emended description of the genus *Thermodesulfovibrio*. *International Journal of Systematic and Evolutionary Microbiology* **2008,** *58*, (11), 2541-2548.
(27) Albertsen, M.; Hansen, L.B.S.; Saunders, A.M.; Nielsen, P.M., A metagenome of a full-scale microbial community carrying out enhanced biological phosphorus removal. *ISME J* **2012,** *6*, 1094-1106.
